# Progress in the Typhoid Conjugate Vaccine Program Rollout Supported by Gavi During the COVID-19 Pandemic and the Path Forward

**DOI:** 10.1093/ofid/ofad042

**Published:** 2023-06-02

**Authors:** Allyson L Russell, Lee M Hampton, Antara Sinha, Francisco J Luquero, Jalaa’ Abdelwahab

**Affiliations:** Vaccine Programs, Gavi, The Vaccine Alliance, Geneva, Switzerland; Vaccine Programs, Gavi, The Vaccine Alliance, Geneva, Switzerland; Vaccine Programs, Gavi, The Vaccine Alliance, Geneva, Switzerland; Vaccine Programs, Gavi, The Vaccine Alliance, Geneva, Switzerland; Vaccine Programs, Gavi, The Vaccine Alliance, Geneva, Switzerland

**Keywords:** antimicrobial resistance, COVID-19 pandemic, immunization programs, typhoid conjugate vaccine, typhoid fever

## Abstract

Gavi supports countries to introduce typhoid conjugate vaccine (TCV) with catch-up campaigns. Available TCVs are highly efficacious, equity-focused, and critical to curbing the expansion of antimicrobial resistance. Four Gavi-supported countries have introduced TCVs since 2018. In the wake of the COVID-19 emergency, momentum is building to scale up TCV introduction worldwide, supported by global partners and Gavi’s funding for improved typhoid diagnostics.

Typhoid conjugate vaccine (TCV) was included in Gavi’s vaccine portfolio during the Vaccine Investment Strategy in 2008, with updates in 2018, following the World Health Organization’s (WHO’s) Strategic Advisory Group of Experts on Immunization (SAGE) recommendation for TCV use [1]. The Gavi Board approved funding to support TCV introductions and to conduct impact evaluations of these introductions in Gavi-eligible countries with the aim of addressing the global burden of typhoid disease and mitigating the growing risk of antimicrobial resistance, recognizing the cost-effective impact of vaccination on morbidity and mortality. (For more detail on the history of Gavi’s investment and the development of WHO pre-qualified TCVs, refer to Soble et al. [2]). However, it took over a decade and significant financial investment from the Bill & Melinda Gates Foundation, among other partners, for efficacious TCVs to be developed and prequalified by WHO, and thereby available to countries to introduce into routine immunization schedules with Gavi support.

Gavi is committed to supporting countries to better characterize and assess the burden of disease due to typhoid and to introducing TCV “in countries with the highest burden of typhoid disease or a high burden of antimicrobial resistant *S.* Typhi,” in accordance with WHO recommendations [1]. Gavi’s current funding guidelines align with these recommendations, which include introduction of a single dose of TCV into routine administration aligned with other routine immunizations at 9 or 15 months, coupled with a single-dose catch-up campaign for children aged 9 months to 14 years. Financial support includes a vaccine introduction grant for routine introduction and vaccine doses co-financed by Gavi and the introducing country, and operational costs for the associated one-off catch-up campaign with vaccine doses fully financed by Gavi. Applications consist of a campaign plan of action, which details the strategies the country will use to reach high coverage during the catch-up campaign, and a new vaccine introduction plan, which specifies the plans for integrating a new vaccine into the routine immunization program (application guidance for vaccine support from Gavi is available in [3]). Countries should plan for ∼12–15 months from the time of application to the launch of the TCV program in-country. Countries can also access Gavi-funded TCV to respond to confirmed typhoid outbreaks. Most recently in December 2021, Gavi's board approved funding to expand the availability and use of fit-for-purpose diagnostic tests for typhoid, among other high-priority pathogens with epidemic potential, in order to assist countries to better define their typhoid burden of disease and use TCVs effectively.

TCV is recognized as an important tool in the fight against antimicrobial resistance and the spread of antimicrobial-resistant strains of *S.* Typhi [4, 5]. Recent analyses demonstrate the acquisition of gene mutations leading to antimicrobial resistance across multiple lineages and an international expansion of these multidrug-resistant (MDR) *S.* Typhi clones, in particular from Asia to Africa, where MDR typhoid is on the rise [6]. Further, the recent emergence and subsequent dominance of extensively drug resistant (XDR) typhoid in Pakistan coupled with the spread of azithromycin-resistant *S.* Typhi strains in other countries in Southeast Asia [6] raises the urgency of expanding prevention measures, including the use of TCV.

TCV is also a critically important vaccine in the equity agenda, given that those who would benefit most from it are typically from underserved communities facing multiple deprivations including limited access to safe water and sanitation. Given the targeted age groups, routine TCV administration and catch-up campaigns can be leveraged to identify infants and adolescents who have missed prior vaccinations and provide an opportunity for catch up of other antigens, such as measles-containing vaccine (MCV) or HPV.

## PROGRESS TO DATE

Since Gavi’s funding window for TCV opened in 2018, Pakistan, Nepal, Liberia, and Zimbabwe became the first countries in Asia and Africa to introduce TCV nationwide. These countries deemed TCV a key intervention to reduce high morbidity and mortality from typhoid, especially with the emergence of XDR strains of *S.* Typhi in both Pakistan and Zimbabwe, which strongly influenced these countries’ decisions to quickly introduce TCV to curb the spread of antimicrobial-resistant *S.* Typhi. Countries that have introduced TCV to date have triangulated data from multiple sources to inform National Immunization Technical Advisory Group (NITAG) decision-making on TCV introduction, including typhoid incidence from sentinel sites, hospital-based data on ileal perforations, and modeled burden of disease data. Importantly, these countries decided to pursue TCV introduction amidst the competing priorities and challenges posed by the coronavirus disease 2019 (COVID-19) pandemic and COVID-19 vaccine rollout, given the impact the vaccine would have on reducing disease transmission and burden in their countries. In numerous other countries, decision-making regarding new vaccine introduction—TCV key among these as one of the newer vaccines available with Gavi support—was put on hold while countries addressed the immediate priority of COVID-19. All 4 countries that introduced TCV thus far included nationwide catch-up campaigns for children up to age 15 years alongside introduction into routine immunization schedules. In total, nearly 50 million children were immunized through these campaigns and protected against typhoid disease, with nationwide survey-based campaign coverage estimates ranging from 63% in Liberia to 88% in Pakistan (weighted average of coverage from phases 1 and 2 [29m target population] out of 3 total phases [35m total target population] planned).

Recent evidence from rigorous studies implemented in a range of geographies including Nepal, Bangladesh, and Malawi suggests a consistent and high level of protection from TCV, with efficacy between 79% and 84% [7–9]. Vaccine effectiveness following TCV introduction has been estimated to be >95% in Pakistan [5, 10] Yousafzai et al. also demonstrated that TCV protected children equally well against all strains of culture-confirmed *S.* Typhi, including XDR strains (97%; 95% CI: 93%–96%) [5], in Hyderabad, Pakistan. The impact of TCV introduction on overall typhoid incidence is currently being assessed, and the results and takeaways from recent introductions, including the impact achieved by the broad-age catch-up campaigns to curtail the spread of MDR and XDR strains, will be published in the near future.

These findings from large-scale vaccine efficacy and effectiveness studies along with successful introduction experiences from early adopter countries are encouraging for other countries that are considering the potential impact of TCV in their contexts. As of July 2022, NITAGs from 6 additional countries have recommended nationwide TCV introduction (ie, Bangladesh, India, Burkina Faso, Kenya, Malawi, and Zambia), and these countries are planning TCV introductions. Multiple additional countries are now engaged in the decision-making process, reviewing evidence on burden, assessing cost-effectiveness, and determining if, where, and how TCV could be used most effectively.

To respond to the anticipated growth in use of TCV globally, the Gavi Vaccine Alliance partners developed a 10-year Roadmap in 2021 [11] to ensure a healthy market for TCV. To date, supply availability has not limited or delayed routine introductions or catch-up campaigns, and partners are working together to maintain this supply security and the sustainability of the market in the mid to long term, coordinated through the Roadmap's action plan.

## LESSONS LEARNED FROM COUNTRY INTRODUCTIONS OF TYPHOID CONJUGATE VACCINE

Early-introducer countries reported a few main challenges along with a few key strategies that led to successful catch-up campaigns and routine immunization launches thus far (unpublished data from Ministry of Health reports and presentations).

The primary challenge these countries experienced in obtaining high coverage during the campaigns was related to lack of information and misinformation on both the timing of the campaign and the vaccine itself, which resulted in hesitancy on the day of vaccination or missing the vaccination campaign date. In many cases, this resulted from the complexity of introducing a new vaccine amidst the COVID-19 pandemic, such as school closures, and ensuring appropriate campaign timing and messaging during the rollout of multiple vaccines simultaneously so as to minimize confusion and spread of misinformation among the public. Integrated campaigns, such as Zimbabwe’s TCV introduction, also cited the challenge of clear and effective communication to the public about multiple vaccinations targeting different diseases and age groups being implemented simultaneously. Early, appropriate, and tailored messaging—especially regarding vaccine safety and campaign timing—that reaches all targeted communities is therefore one of the most critical factors for success.

Other strategies for success included strong government commitment and coordination among stakeholders, private sector engagement including relying on private practitioners as key influencers to generate demand, using prior campaign experiences to improve microplanning exercises, and leveraging technology for real-time monitoring during the campaign. Early and strong coordination and advocacy with the Department of Education, including school health coordinators, and with parents to generate demand and ensure consent was given prior to the vaccination day were critical to achieving high levels of vaccination coverage. Effective community engagement and social mobilization, informed by focus group discussions with pediatricians, parents, and health workers, paired with strong health worker interpersonal skills, were also reported as critical to the success of the introductions and campaigns.

More broadly, countries are beginning to use the TCV catch-up campaigns as a means to achieve other vaccination-related goals, such as the identification of under- and nonvaccinated children and reaching them with needed vaccines. Countries have used the TCV catch-up campaign as an opportunity to conduct mop-up sessions for other antigens and national immunization weeks and integrate with planned vaccination sessions in schools. In Nepal, vaccinators were explicitly reviewing child vaccination records, identifying those missing doses, providing counseling to caregivers on the routine immunization schedule, and connecting these children to the routine immunization system for catch-up vaccination. Rapid convenience monitoring estimated that 11.5% of children aged 15–49 months were missing 1 MCV dose, and 1.5% had not received any MCV (unpublished data, presentation by Nepal MOH, Sept 2022). The campaigns also provided the opportunity for health system manager and health care worker capacity building in conducting a wide-age injectable campaign, which is valuable as an increased number of life course vaccines become part of routine immunization. In addition, Pakistan's phased approach to introduction allowed the country to incorporate takeaways from the earlier campaigns into subsequent phases, in particular how to identify groups that were likely to be missed by vaccination campaigns based on historical experience and conduct specific outreach for these groups. Surveillance data on unvaccinated persons presenting with typhoid fever also provided an opportunity to identify gaps in immunity, both in geographic areas and in certain populations or individuals, and to use this information to inform future vaccination strategies.

## ANTICIPATED FUTURE GROWTH OF TCV PROGRAM INCLUDING USE OF DIAGNOSTICS FOR IMPACTFUL DECISION-MAKING

Over the next 5 years, vaccine demand is anticipated to increase as new evidence emerges about the impact of TCV in countries that have already introduced the vaccine and more countries assess their typhoid burden and make evidence-based decisions on the use of TCV. While several countries delayed the decision-making process due to competing priorities during the intensive period of the COVID-19 response, strategic planning discussions including new vaccine introduction plans have now resumed. Experience with previous new vaccine programs supported by Gavi suggests that countries’ demand for TCV is likely to increase at an accelerated pace compared with the first four years of the program. As an example of new program growth trajectory, 15 of 57 countries eligible for Gavi new vaccine support had already used a second dose of measles-containing vaccine (MCV2) when Gavi funding for MCV2 began in 2007. By 2011, only 4 additional countries introduced MCV2, for a total of 19 countries; however, by 2015, that number rose to 41 countries [12]. The Gavi Vaccine Alliance and other partners including the Centers for Disease Control and Prevention (CDC), the Bill & Melinda Gates Foundation, and PATH are committed to supporting countries’ decision-making, prioritization, and vaccine introduction efforts in order to accelerate the pace of introductions and maximize the public health benefit of TCV worldwide.

Nevertheless, the challenges countries face to rapidly and accurately identify typhoid infection with current diagnostic tools and the resulting lack of reliable burden data continue to substantially hamper decision-making around the use of typhoid vaccine in routine immunization programs. Detailed and accurate typhoid incidence data guide country decisions on the introduction of TCV in routine immunization and targeting of TCV in routine immunization and catch-up campaigns, detection and response to typhoid outbreaks, and identification and correction of gaps in TCV delivery following introduction into routine immunization. As articulated by the WHO, laboratory-based bacterial blood culture is currently the mainstay for confirmation of typhoid cases due to the limitations of other tools [13], such as the low accuracy of currently available point-of-care tests [14]. However, current blood culture testing also has limitations, including low sensitivity and logistical and technical complexities that can make it difficult to scale up and sustain in resource-limited settings [15]. As a result, 16 countries eligible for Gavi typhoid vaccine support have not had published public reports of typhoid burden based on reliable, blood culture–confirmed surveillance data since at least 1995 [16]. For many of the countries that do have reliable surveillance data, those data may not be generalizable across the entire country as they come from sentinel sites that cover only relatively small geographic areas [17]. Gavi’s current guidance for applications for TCV introduction acknowledges these challenges and advises countries to triangulate available data from sentinel surveillance, research studies, laboratories, and, where needed, regional and modeled data to develop the rationale for TCV introduction (guide for data for Gavi TCV applications is available in [18]).

Since the limitations of current typhoid tests are a major factor impairing typhoid surveillance, improved, fit-for-purpose typhoid diagnostic tests could greatly aid country efforts to increase the availability of reliable typhoid incidence data [19]. As a result, the WHO, UNICEF, the Foundation for Innovative New Diagnostics (FIND), the CDC, the Bill & Melinda Gates Foundation, and the Gavi Secretariat are implementing a multifaceted approach to facilitate the availability of such tests in the market and to countries for utilization for burden estimation and vaccine-related decision-making [20]. First, Target Product Profiles (TPPs) are under development by a WHO expert group to indicate the characteristics tests should have to warrant procurement by international organizations for typhoid surveillance. Given the range of information relevant to decisions on targeting of TCV, including overall incidence of typhoid and incidence of antimicrobial-resistant typhoid, multiple types of tests may be needed. Guided by these TPPs, the WHO will assess typhoid test kits submitted by manufacturers, drawing on information submitted by manufacturers as well as independent laboratory evaluations of typhoid test kits. Test kits assessed as satisfactory will be eligible for pooled procurement organized by UNICEF. Once relevant tests are available, guidance for their use and distribution will be published and is likely to draw upon modeling studies and in-country deployment pilot projects. All countries eligible for vaccine support through Gavi will be able to apply for Gavi typhoid diagnostic test kit procurement funding support to facilitate testing to inform TCV decision-making, with the condition that the countries eventually assume financial responsibility for the continued procurement of such test kits.

## CONCLUSIONS

In summary, momentum is building for TCV introduction in Gavi-supported countries across Asia and Africa after delays in planned introductions as a result of the COVID-19 pandemic. This is bolstered by recent evidence demonstrating consistently high efficacy and effectiveness of WHO-prequalified TCVs and a growing urgency to slow the spread of MDR and XDR strains of typhoid. New support from Gavi to bring fit-for-purpose diagnostic tests to market and made available for countries will improve countries’ ability to assess the true burden of typhoid disease and make evidence-based decisions on TCV introduction. TCV supply is expected to be able to meet this growing demand, and a cross-Alliance team is working with a market-shaping roadmap to ensure the supply security and sustainability of the TCV market. As countries consider and plan deployment of TCV, it will be critical to maintain the focus on equity, ensuring those most in need and most vulnerable are reached and routinely have access to not only TCV but all the immunizations and health services that they need.
